# Extracellular Matrix and Cancer: An Intricate Affair

**DOI:** 10.3390/ijms241813969

**Published:** 2023-09-12

**Authors:** Maurizio Mongiat

**Affiliations:** Department of Research and Diagnosis, Division of Molecular Oncology, Centro di Riferimento Oncologico di Aviano (CRO) IRCCS, 33081 Aviano, Italy; mmongiat@cro.it; Tel.: +39-0434-659516

In complex multicellular eukaryotes, the extracellular matrix (ECM) is an essential component of the organism, not only providing structure to the tissues, but also granting cellular cooperation through the engagement of an intricate crosstalk between all cell types.

In fact, the ECM takes part in many processes, including cell differentiation and homeostasis, as well as the immune response. For this strategic role, the ECM's expression and complex composition is tightly regulated; however, this equilibrium is lost under pathological conditions. For instance, cancer is characterized by extensive ECM remodeling thanks to abnormal proteolytic enzyme activation; furthermore, altered ECM expression has been linked to tumor onset. Of note, aberrant ECM composition not only affects tumor progression directly, impinging on the tumor cell growth and viability of, but also indirectly, impacting the function of other microenvironmental cell types, including cancer-associated fibroblasts and immune cells. 

In the latest few years, interest in the study of the microenvironment and its components has been revitalized. This also due to the fact that ECM remodeling leads to the liberation of fragments that could be potentially be exploited as putative predictive/prognostic biomarkers.

The subjects dealt with in this Special Issue represent an exciting glimpse of all these aspects.

To get to the heart of the studies, Victoria Catalán and colleagues evaluate the role of dermatopontin (DPT) in obesity-associated colon cancer development [1]. The authors demonstrate that the circulating levels of DPT increase in obese patients; however, they significantly decrease during colon cancer development. Through in vitro studies the authors demonstrate that DPT expression by tumor cells is positively regulated by inflammation, and that anti-inflammatory cytokines halt its expression. In addition, the authors provide further evidence to the concept that changes in the expression of a given ECM molecule eventually lead to widespread changes in ECM composition and microenvironment. They prove that DPT elevates the expression of the collagen type I alpha1 chain, collagen type V alpha 3 chain, Tenascin-C and VEGF-A, and is also associated with reduced decorin expression. Importantly, colon cancer cell treatment with conditioned media from adipocytes isolated from obese patients enhanced the DPT expression, highlighting the importance of this ECM molecule in obesity-associated colon cancer onset.

In another original study, Veronica De Paolis and colleagues highlight the link between ECM and the tumor immune response [2], which is gaining increasingly more interest also in the light of the promising employment of immunotherapy. In fact, the infiltration, differentiation and activation of immune cells is profoundly influenced by the microenvironment. In this study the authors explore the role of perlecan, also known as heparan sulfate proteoglycan 2 (HSPG2). Increased levels of this proteoglycan are associated with poor breast cancer patient prognosis, and the results provided by the authors suggest that tumor-associated macrophages (TAMs) represent an important source of this molecule. In particular, high HSPG2 deposition was induced by the tumor-promoting M2 macrophages and associated with the increased expression of the canonical and non-canonical NF-κB signaling pathway components RelA (p65) and NFKB2 (p52). Importantly, HSPG2 expression was also dependent on tumor stiffness. This study further highlights the interconnection between ECM, the tumor microenvironment, and the immune response in addition to their reciprocal influence, which is essential in determining tumor progression and outcome.

In an interesting review, Aleksandr Popov and colleagues nicely summarize an important aspect in the field of matrix biology [3], i.e., the possibility to employ matrikines, biologically active fragments arising from the degradation of collagens and glycosaminoglycans, as potential molecules to be exploited for cancer treatment. In fact, there are many advantages in the putative use of these molecules, including the fact that as endogenous molecules, there is no potential risk of side effects, they are relatively small and display excellent cellular diffusion and permeability, and they can be employed in relatively low concentrations. The ECM affects many aspects of cell physiology; thus, the number of matrikines able to be exploited in this context is potentially vast. The authors take into account matrikines deriving from collagen, elastin, haluronan and laminin, also highlighting the importance of metalloproteinase involved in their generation. In this view, the ECM may represent an inexhaustible source for the development of new less toxic and more efficient drugs for cancer treatment.

The following review by Anastasia Strokotova and Elvira Grigorieva deals with the use of glucocorticoids (GCs) in the treatment of many diseases, including cancer, and the considerable side effects that characterize their long-term employment [4]. The molecular basis responsible of these unwanted effects are poorly understood; however, increasing evidence suggest that they may, at least in part, be ascribed to their impact on the ECM and, in particular, proteoglycans (PGs) and glycosaminoglycans (GAGs). The authors review the PG expression and GAG content in relation to GC treatment in different normal tissues and cells, as well as in tumors. This review further emphasizes the concept that ECM is not a static component; on the contrary, it is continuously remodeled by a plethora of mechanisms, thus profoundly affecting disease onset and progression.

Finally, Kena Song and colleagues review the importance of collagens and their remodeling in affecting cancer progression [5]. Once again this study highlights the importance of ECM remodeling in affecting cancer progression and pinpoints collagens as key molecules in this context. While they represent a physical barrier under normal conditions, upon rearrangement in the tumor microenvironment, they have been shown to paradoxically aid tumor progression. The authors underline the importance of these components in many aspects, characterizing tumor progression, such as tumor stiffness, angiogenesis and tumor cell invasion. As a ‘side effect’ of collagen remodeling, the generated collagen fragments released in the patients' serum may represent promising prognostic/predictive biomarkers that are easily accessible by liquid biopsy. This evidence further confirms the importance of studying the microenvironment to develop new clinical tools to improve the management of cancer patients.

In conclusion, the ECM’s affairs in the tumor microenvironment are intricate and change drastically during tumor progression due to its remodeling. As a consequence, these changes not only affect tumor progression, but also the fragments, thus creating the possibility to develop new biomarkers and/or drugs to improve the treatment of cancer patients. The reviews and original research articles published in this Special Issue are representative of these archetypal concepts, and I am confident you will enjoy reading the aforementioned studies.
